# Bronchoplasty using continuous suture in complete monitor view: a suitable method of thoracoscopic sleeve lobectomy for non-small cell lung cancer

**DOI:** 10.1186/s12957-016-0895-4

**Published:** 2016-04-30

**Authors:** Feng Shao, Zhengcheng Liu, Yanqing Pan, Hui Cao, Rusong Yang

**Affiliations:** Department of Thoracic Surgery, Nanjing Chest Hospital Affiliated to Southeast University, Nanjing, 210029 China; Department of Thoracic Surgery, Nanjing Chest Hospital, Nanjing, Jiangsu Province 210029 China

**Keywords:** Continuous suture, Bronchoplasty, Video-assisted thoracic surgery (VATS), Non-small cell lung cancer

## Abstract

**Background:**

Our study aims to determine the value of bronchial anastomosis using complete continuous suture.

**Methods:**

Six patients diagnosed with central lung carcinoma who were candidates for right-sided sleeve lobectomy and underwent sleeve resection of the right upper lobe by thoracoscopic surgical procedure.

**Results:**

The mean surgical time was 182 min (range, 110 to 260 min). The mean time of bronchial anastomosis was 49 min (range, 18 to 76 min). The mean bleeding was 110 mL (range, 50 to 260 mL). Median chest tube drainage was 305 mL (range, 200 to 600 mL). No perioperative deaths or major complications occurred. The postoperative bronchoscopy confirmed no stenosis. The mean follow-up time was 19.2 months (range, 7 to 34 months), and six patients were alive.

**Conclusions:**

Bronchial anastomosis using complete continuous suture may be a suitable method in thoracoscopic sleeve lobectomy.

## Introduction

Thoracotomy is the traditional way to perform a bronchial sleeve lobectomy for non-small lung cancer (NSCLC), but it also can be performed by video-assisted thoracic surgery (VATS). The key point of thoracoscopic sleeve lobectomy is to finish bronchial anastomosis in the complete monitor view. The traditional interrupted suturing techniques emphasize on the security and less anastomotic site ischemia, while the continuous suturing techniques will result in less suture tangling and may be quicker. There is no evidence of which is the better between interrupted and continuous suturing in bronchial anastomosis. Since the first case was reported [1], we have performed six cases of NSCLC with bronchial anastomosis using complete continuous suture which we believe to be a suitable method in thoracoscopic sleeve lobectomy. The following section details our method.

## Material and methods

### Clinical data

From April 2012 to October 2014, six patients diagnosed with central lung carcinoma who were candidates for right-sided sleeve lobectomy and satisfied the criteria in Table 1 were eligible for the video-assisted thoracic surgery (VATS) approach. Preoperative evaluation included a computed tomographic (CT) scan of the chest, positron emission tomographic (PET/CT) scan, fiberoptic bronchoscopy, and pulmonary function tests with diffusion capacity. Preoperative electrocardiogram and heart function were no contraindication. Maximum tumor size bigger than 5 cm, main bronchi or vessel invasion, enlargement or calcification of mediastinal and hilar lymph nodes in CT and PET/CT, and heavy adhesion of pleural cavity were the exclusion criteria. Fiberoptic bronchoscopy confirmed exophytic tumor obliterating the distal part of the right main bronchus and infiltrating orifices of both the upper and the intermedius bronchus. The patients’ clinical data are shown in Table 2.

**Table 1:** Criteria for thoracoscopic sleeve lobectomy for primary lung carcinoma

Maximum tumor size 5 cm, location above the segment bronchi, necessary for sleeve lobectomy
No evidence of bronchi and vessel invasion, no direct invasion to the surrounding organs requiring reconstruction
No enlargement or calcification of mediastinal and hilar lymph nodes in CT and PET/CT
No heavy adhesion of pleural cavity, suitable for VATS
Patient and family agreed to procedure

**Table 2:** Patient characteristics and histology

No.	Age	Sex	Tumor size (mm)	Histology	Pre-op pulmonary functional test			Clinical stage	Neoadjuvant treatment
					FEV1%	FEV1/FVC%	DLCo%		
1	64	Male	35	Squamous cell	88.4	80.3	63.7	T2N0M0, IB	No
2	60	Female	20	Carcinoid	93.1	83.7	67.2	T2N0M0, IB	No
3	59	Female	28	Adenocarcinoma	85.4	77.8	59.3	T2N0M0, IB	No
4	67	Male	35	Squamous cell	93.9	82.6	65.1	T2N1M0, IIB	No
5	73	Male	42	Squamous cell	62.5	60.4	50.9	T2N1M0, IIB	No
6	49	Female	30	Adenocarcinoma	76.9	69.3	61.4	T2N0M0, IB	No

## Surgical technique

All procedures performed in this study involving human participants were in accordance with the ethical standards of the institutional and/or national research committee in our hospital and with the 1964 Helsinki declaration and its later amendments or comparable ethical standards. For this type of study, formal consent is not required. Six of them underwent sleeve resection of the right upper lobe by thoracoscopic surgical procedure. We placed the patient in a left lateral decubitus position. A 1-cm port on the middle axillary line in the seventh intercostal space was the first port used primarily for thoracoscopy. The second and the third ports, 2 and 1.5 cm, respectively, on the anterior axillary line in the fourth intercostal space and the posterior axillary line in the ninth intercostal space, were used for manipulation of the lung.

**Fig. 1:**
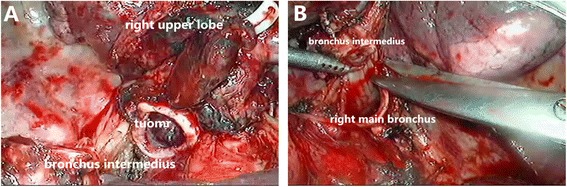
**a** The bronchus intermedius were circumferentially dissected 1.0 cm away from the right upper bronchi. **b** Dissection of the right main bronchi 1.5 cm away from the right upper bronchus

The right upper lobe vein was divided with a 45-mm vascular stapler while taking care to avoid injury to the middle lobe vein. The truncus anterior branch of the right pulmonary artery and two branches of the posterior ascending artery were then divided in a similar manner with hemolock. After division of the arterial supply to the right upper lobe and identification of the arteries supplying the middle and lower lobes, the major and minor fissures were completed with a 60-mm linear stapler. Following resection of hilar lymph node and inter-lobes lymph node, the bronchial sleeve resection may begin. The bronchus intermedius were circumferentially dissected 1.0 cm away from the right upper bronchi, then mobilization of the right upper lobe, dissected the right main bronchi 1.5 cm away from the right upper bronchi (Fig. 1). With care being taken not to devascularize the airway. Frozen sections of the cut ends of the right main bronchus and the bronchus intermedius were negative of tumor infiltration as confirmed pathologically during surgery.

**Fig. 2:**
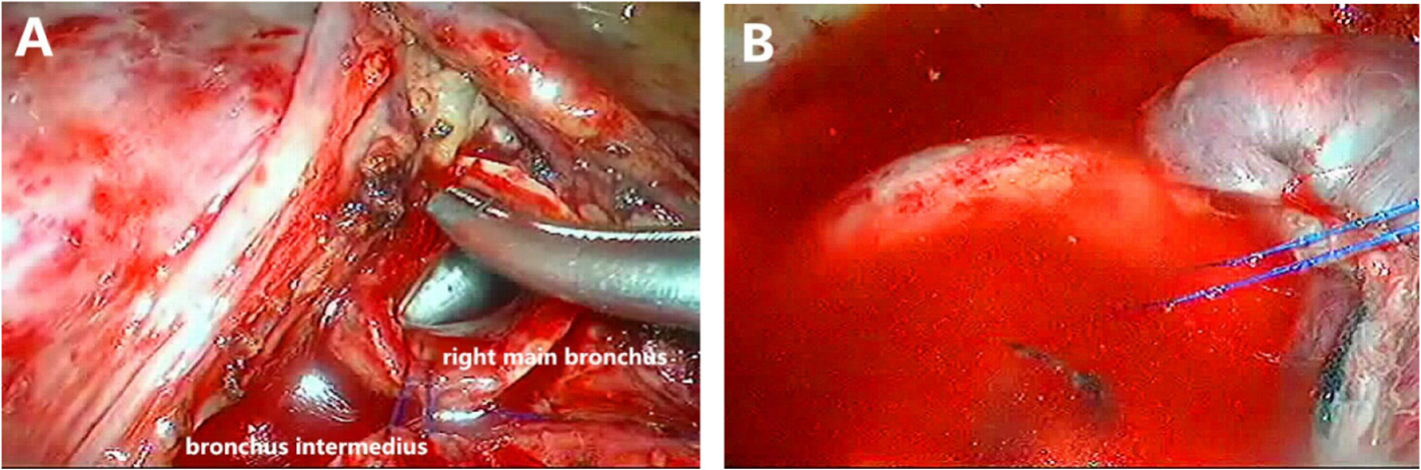
**a** The end-to-end anastomosis of the intermediate and mainstem bronchus. **b** Anastomosis was tested no air leak for pneumostasis by submerging it under saline and inflating the lung to a pressure of 20 cm of water

Dividing the inferior pulmonary ligament was performed to release the tension of airway anastomosis. The end-to-end anastomosis begun by placing traction sutures at both of the cartilaginous-membranous junctions to help approximate the intermediate and mainstem bronchi. We used 3-0 prolene (Ethicon, Somerville, NJ) continuous sutures to close the membranous and bronchial cartilage (from the posterior to anterior) with the help of an endoscopic knot pusher. The sutures were dragged tight at one time, anastomosis was tested for pneumostasis by submerging it under saline and inflating the lung to a pressure of 20 cm of water (Fig. 2). We finished by tying the knots under camera without using any tissue flap to protect the anastomosis. Postoperative bronchoscopy is then performed to clear the airways of blood and secretions before extubation.

## Results

The mean surgical time of the six patients was 182 min (range, 110 to 260 min). The mean time of bronchial anastomosis was 49 min (range, 18 to 76 min). The mean bleeding in operation was 110 mL (range, 50 to 260 mL). Median chest tube drainage was 305 mL (range, 200 to 600 mL). There were no intraoperative or postoperative deaths. The patients had no intraoperative bronchial air leak. No major complications occurred. The mean hospital stay was 9.2 days (range, 7 to 12 days). The postoperative bronchoscopy 1 month after surgery confirmed no stenosis. The mean follow-up time was 19.2 months (range, 7 to 34 months), and six patients were alive (Table 3).

**Table 3:** Intraoperative and postoperative variables

No.	Operating time (min)	Suturing time (min)	Bleeding (mL)	Drainage (mL)	Hospital stay (day)	Pathological stage	Follow-up (month)
1	260	76	100	250	8	T2N0M0, IB, R0	34
2	210	60	50	200	11	T2N0M0, IB, R0	27
3	180	64	70	320	9	T2N1M0, IIB, R0	22
4	190	42	260	600	12	T2N2M0, IIIA, R0	16
5	140	33	100	260	8	T2N1M0, IIB, R0	9
6	110	18	80	200	7	T2N0M0, IB, R0	7

## Discussion

With improvements in thoracoscopic competency, greater exchange of knowledge and techniques, and advances in equipment, increasing number of medical centers are able to perform video-assisted thoracic surgery sleeve lobectomy and even double sleeve lobectomy for central lung cancer [2]. Technically, the most important and difficult part of thoracoscopic sleeve lobectomy is to complete bronchial anastomosis in the monitor view. Interrupted suturing techniques have potential advantages of less anastomotic site ischemia and security, while continuous suturing techniques will result in less suture tangling and may be quicker. It seems impossible to have a meaningful comparison of clinical outcomes between the different anastomotic approaches for thoracoscopic sleeve lobectomy because of the relatively low case numbers [3].

Some of reports describe the VATS approach using interrupted sutures in anastomosis, especially in bronchus cartilage reconstruction [4, 5]. The end-to-end anastomosis can also be performed by complete continuous suture. Some authors believe that anastomosis with continuous sutures is thought to be useful for bronchoplasty in thoracoscopic surgical procedures [6]. We think that the key point of sutures in bronchial anastomosis in VATS is to avoid tangling the ends of the untied ends. Since 2003, we have used continuous suture to complete both membranous bronchus and cartilage anastomosis at one time through thoracotomy. Consequently, as we accumulated experience, we were able to perform sleeve lobectomy with VATS. Complete continuous suture was an ideal way in thoracoscopic sleeve lobectomy with only one 3-0 prolene to avoid tangling the ends of the untied ends. With an endoscopic knot pusher, every suture could be pushed near the bronchus, it was quite clear to adjust for any size discrepancy between the proximal and distal airways with precise suture placement along the circumference of the anastomosis (Fig. 1). Besides, the tension could be carefully adjusted with a sliding knot-pushing instrument. Only by dragging the sutures tight, we can perform air leakage test, and tie the knots while placing the sutures to prevent them from tangling (Fig. 2). With the increasing number of cases, our techniques of continuous suture were more skillful. The time of operation and the time of the bronchial anastomosis were shortened.

## Conclusion

VATS sleeve lobectomy becomes more popular with acceptable morbidity and mortality as well as short length of stays, and a viable choice for some patients with central lung cancer. We believe that complete continuous suture should be a suitable way in thoracoscopic sleeve lobectomy which can be convenient with a clear operative view.

## Ethical approval statement

All procedures performed in this study involving human participants were in accordance with the ethical standards of the institutional and/or national research committee in our hospital and with the 1964 Helsinki declaration and its later amendments or comparable ethical standards. For this type of study formal consent is not required.
